# A pulmonary mass with invasion into the heart

**DOI:** 10.1186/1477-7800-5-24

**Published:** 2008-10-17

**Authors:** Anne Marie McLaughlin, Rory A O'Donnell, Siobhan Nicholson, Joseph Keane, Vincent K Young

**Affiliations:** 1 Department of Respiratory Medicine, St James Hospital, Dublin 8, Ireland; 2 Department of Histopathology, St James Hospital, Dublin 8, Ireland; 3 Department of Cardiothoracic Surgery, St James Hospital, Dublin 8, Ireland

## Abstract

We describe the case of a 58 year old woman who presented with bronchial atypical carcinoid found at surgery to invade the left atrium along the pulmonary veins. A right pneumonectomy and removal of a portion of the left atrium was performed. The patient made an excellent post operative recovery. Three years later she presented in acute respiratory failure secondary to local recurrence. This is first case described in which recurrence after resection of bronchial carcinoid metastatic to the heart is described.

## Background

**Figure 1:**
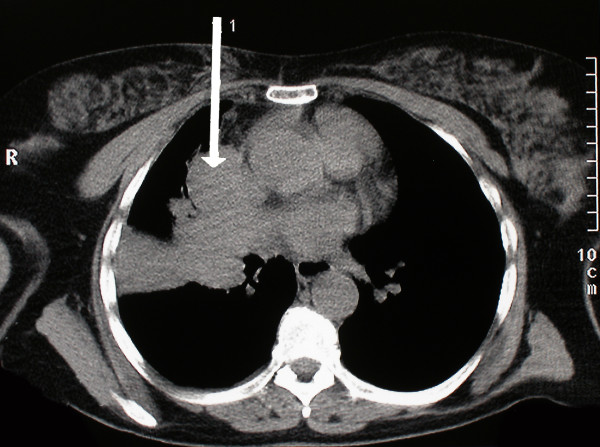
Axial CT image demonstrating the right hilar mass extending into the right middle lobe and distorting the outline of the right and left atria with CT guided percutanous biopsy of the mass.

**Figure 2:**
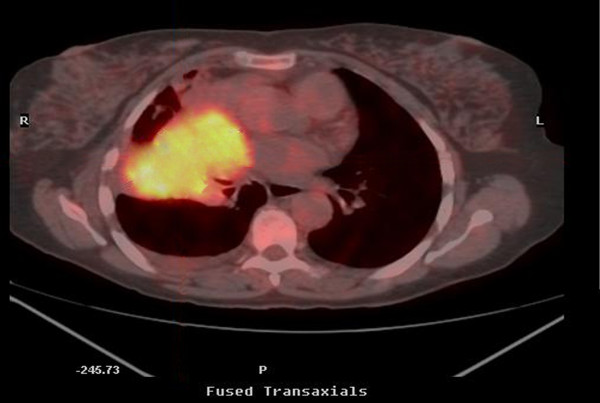
Axial fused PET-CT demonstrating FDG accumulation in a right middle lobe mass.

**Figure 3:**
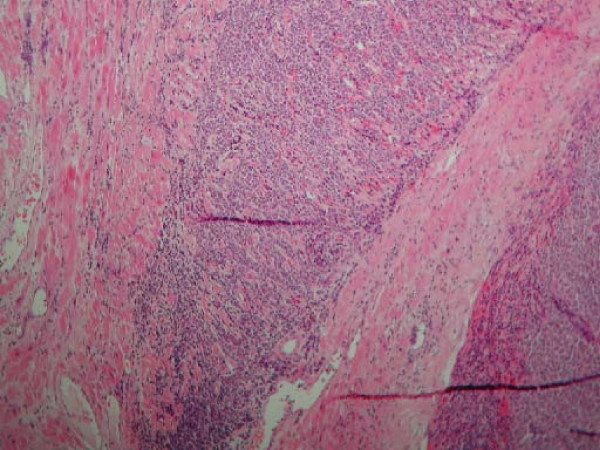
Hematoxylin and Eosin stain, high power view of carcinoid infiltrating atrial muscle.

**Figure 4:**
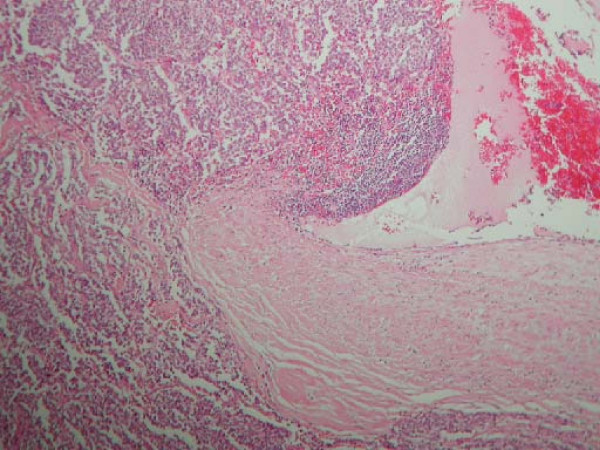
A High power view of carcinoid invading pulmonary artery wall.

A 58 year old woman, who had never smoked, presented with a two week history of chest pain, dyspnoea on exertion and dry cough. She had no symptoms of wheezing or flushing and had no previous medical history. Flexible bronchosocpy demonstrated a narrowing of the anterior segment of the right middle lobe. Computed tomography, at which time percutaneous biopsy was performed, demonstrated a mass at the level of the right hilum extending into the right middle lobe and indenting both the left and right atria (figure 1). Positron Emission Tomography/Computed Tomography (PET/CT) confirmed a large right hilar mass with a Standard Uptake Value (SUV) of 8 (figure 2). Following a multidisciplinary meeting, the decision was taken for her to undergo surgery. A right posterolateral thoracotomy was performed and the mass was identified. The pericardium was opened and the mass seen to invade the left atrium along the pulmonary veins. A right pneumonectomy was performed in addition to removal of a portion of the left atrium using a large vascular clamp and under sewing. The pericardium was closed with a patch of Malex mesh. Histology of the resected pulmonary artery and left atrium are shown (Figure 3 and 4).

Histology confirmed a 8 1/2 cm atypical carcinoid with direct involvement of two perihilar lymph nodes, the pulmonary artery and left atrium, the final staging was T4N1M0. The patient made an excellent post-operative recovery. However, three years following the initial surgery she represented with a two week history of rapidly worsening dyspnoea and hoarseness. She progressed to respiratory failure and required intubation and ventilation. Laryngoscopy demonstrated paralysis of the right vocal cord. CT thorax showed a tumour encasing the distal trachea and left main bronchus and extending into the mediastinum. Palliative stenting of the trachea and left main bronchus was performed. However she continued to deteriorate and develop signs of superior vena caval obstruction. She was subsequently referred for palliative care.

## Discussion

Bronchial carcinoid tumors which account for 1 to 2% of all primary lung malignancies, are part of the spectrum of neuroendocrine lung neoplasia which arise from Kulchitzky cells and includes typical and atypical carcinoid, large cell neuroendocrine carcinoma (LCNEC), and small cell lung cancer (SCLC). Unlike typical carcinoids, atypical carcinoids are associated with a history of cigarette smoking, (83%–94% of cases) and occur more often in men (2:1), the mean presenting age is 46 years with a wide age range [1].

Approximately 85% of bronchial carcinoids occur centrally and manifest with symptoms of bronchial obstruction including wheeze, hemoptysis, cough, chest pain, and recurrent infections. 15% occur peripherally, and are usually clinically silent. Carcinoid syndrome occurs in only 2–7% of cases and is usually associated with advanced metastatic disease [2]. Pulmonary carcinoid may also occur rarely as part of Multiple Endocrine Neoplasia type 1 (MEN 1), associated with neoplasia of pituitary, pancreas and parathyroid.

Radiographic staging of bronchial carcinoids is difficult. The sensitivity of 18 fluorodeoxyglucose (FDG)-PET for diagnosis of pulmonary carcinoids is believed to be reduced due to the low metabolic activity and slow growth of carcinoid tumours. One series demonstrated PET sensitivity of only 14.2%, this led many to believe PET is not effective for diagnosis of pulmonary carcinoid tumours [3]. More recently increased FDG uptake has been reported in atypical carcinoid. Carcinoid tumours have high numbers of somatostatin receptors, which allow scintigraphic imaging with the radiolabeled somatostatin analogue, octreotide. Accordingly, octreotide scanning has been found to be helpful for detecting occult tumours, particularly those with para-neoplastic symptoms.

Metastatic spread associated with atypical carcinoids has been previously described. One case of bronchial carcinoid directly invading the pericardium [4]. and two cases invading the myocardium [5,6]have previously been described in addition there has been a case report of myocardial metastases occurring 8 years following initial resection [7]. Our case is the first described in which there was local recurrence following surgical resection.

Surgery is the preferred approach to treatment for patients whose overall medical and pulmonary condition is adequate. For exceptional cases where the lesion is entirely intraluminal, bronchoscopic resection may be an alternative. Radiation therapy can achieve symptomatic palliation for patients with metastatic/unresectable malignant carcinoid tumors, and it is well tolerated. A long period of follow up is suggested in view of the incidence of late recurrence, illustrated by this case.

## Authors' contributions

AMMcL wrote the manuscript, RO'D and JK were pulmonary physicians for the patient, SN provided the histological figures and VK oversaw the cardiothoracic surgery.
